# The pancreas responds to remote damage and systemic stress by secretion of the pancreatic secretory proteins PSP/regI and PAP/regIII

**DOI:** 10.18632/oncotarget.16282

**Published:** 2017-03-16

**Authors:** Theresia Reding, Cristian Palmiere, Clinsyjos Pazhepurackel, Marc Schiesser, Daniel Bimmler, Andrea Schlegel, Ursula Süss, Sabrina Steiner, Leandro Mancina, Gitta Seleznik, Rolf Graf

**Affiliations:** ^1^ Department of Visceral and Transplantation Surgery, University Hospital Zurich, Zurich, Switzerland; ^2^ Department of Legal Medicine, Lausanne, Switzerland

**Keywords:** sepsis, acute phase reaction, regenerating proteins, pancreas, intestine

## Abstract

**Introduction:**

In patients with infection and sepsis serum levels of Pancreatic Stone protein/regenerating protein I (PSP) are highly elevated. The origin of PSP during these conditions is presumably the pancreas, however, an intestinal origin cannot be excluded. Similarly, pancreatitis-associated protein (PAP) was identified in the pancreas. These proteins were also localized in intestinal organs. Here we aim to elucidate the bio-distribution of PSP and PAP in animal models of sepsis and in healthy humans.

**Results:**

PSP and PAP responded to remote lesions in rats although the pancreatic response was much more pronounced than the intestinal. Tissue distribution of PSP demonstrated a 100-fold higher content in the pancreas compared to any other organ while PAP was most abundant in the small intestine. Both proteins responded to CLP or sham operation in the pancreas. PSP also increased in the intestine during CLP. The distribution of PSP and PAP in human tissue mirrored the distribution in the murine models.

**Materials and methods:**

Distribution of PSP and PAP was visualized by immunohistochemistry. Rats and mice underwent midline laparotomies followed by mobilization of tissue and incision of the pancreatic duct or duodenum. Standard cecum-ligation-puncture (CLP) procedures or sham laparotomies were performed. Human tissue extracts were analyzed for PSP and PAP.

**Conclusions:**

The pancreas reacts to remote lesions and septic insults in mice and rats with increased PSP synthesis, while PAP is selectively responsive to septic events. Furthermore, our results suggest that serum PSP in septic patients is predominantly derived through an acute phase response of the pancreas.

## INTRODUCTION

Pancreatic Stone Protein (PSP/regI) [1] and Pancreatitis-Associated Protein (PAP/regIII) [2, 3] have been identified several decades ago. Initially, their role was associated with pathological changes in the pancreas due to acute and chronic pancreatic inflammation [4]. Over time several hypotheses concerning their function were proposed [5, 6]. Briefly, after identification of PSP in the 1970s, it was assumed to inhibit stone formation based on its association with pancreatic depositions [7]. In the late 1980s PSP was independently found in islets [8, 9] and, due to its up-regulation after organ injury, was thought to have a regenerative effect and thus was named islet-derived regenerating protein (reg). During this early period, PSP was also named pancreatic thread protein, lithostatin and regenerating protein I, while PAP was renamed regenerating protein III.

In the early 1990s PAP was identified during acute pancreatitis in rats and in humans [2, 3]. Both PSP and PAP were demonstrated to promote bacterial aggregation [10]. Further studies showed the presence of PSP and/or PAP in the brain [11, 12], the stomach [13], and intestine [14]. However, for most organs the function of PSP and PAP remains obscure.

Serendipitous observations in animal experiments led us to demonstrate PSP as an indicator of systemic stress in the rat [15]. To validate the clinical relevance for the PSP response we analyzed a cohort of trauma patients. We were able to demonstrate that serum PSP rises early after trauma in those patients that later develop infection and sepsis [16]. In contrast, well known parameters of general inflammation such as procalcitonin or Interleukin-6 were less sensitive. Other studies on sepsis [17], peritonitis [18], ventilator associated pneumonia [19, 20], or in pediatric patients [21, 22] demonstrated a predictive value for outcome.

Current research on the therapy of septic complications demonstrates that there is no commonly accepted algorithm for the treatment of patients and empirical antibiotic application varies significantly among centers [23]. Although recognized as a cornerstone for early treatment of sepsis [24], broadband antibiotic treatment may lead to an increase in multi-resistant bacteria. Biomarkers helping to identify early inflammation and to better assess patients and guide the decision for an adequate and targeted therapy are therefore still needed [25]. Due to the complexity of physiological reactions a ‘one fits all’ biomarker does not exist, however, panels of biomarkers covering different diseases might be more useful [26]. Algorithms weighing the contribution of each biomarker require insights into regulation, functional mechanism, and reaction to specific conditions. Efficacy of PSP as such a biomarker is being evaluated in various clinical studies. A number of questions remain open: which organs mainly produce PSP and PAP, which organs are responsive to infected or septic conditions, and how can we explain the appearance of PSP in blood under both, healthy and pathological conditions.

Here we show the bio-distribution of PSP and PAP in rats, mice, and humans. In animal experiments we show the reaction of various organs to tissue damage and a septic challenge.

## RESULTS

### Bio-distribution of PSP and PAP in the healthy rat

**Figure 1:**
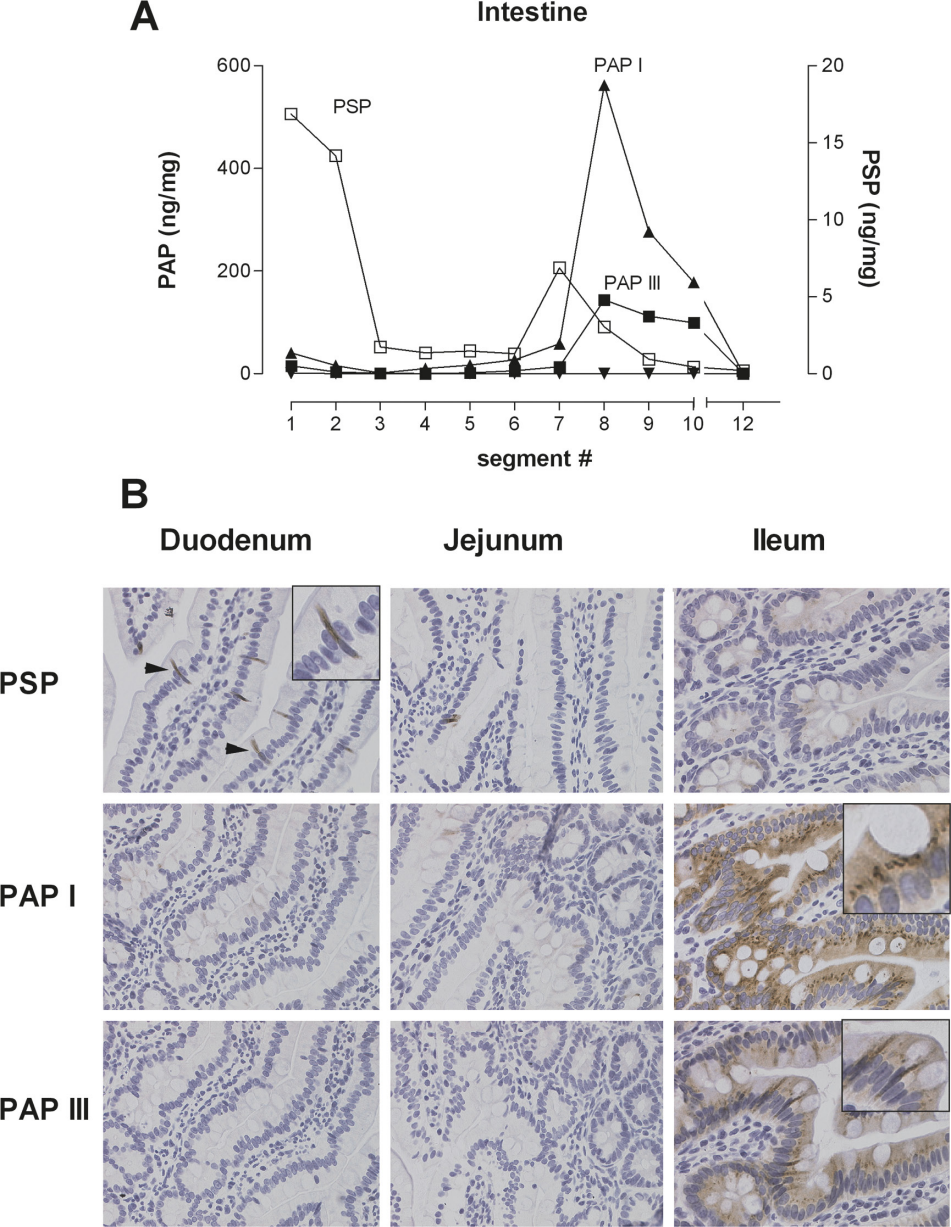
Representative example of tissue distribution of PSP and PAPI, II, III in the rat intestine (**A**) Ten intestinal segments and the colon were collected and protein was extracted. PSP and PAP was quantified by isoform specific ELISA, expressed as ng/mg total protein. PAP refers to the left y-axis, PSP to the right x-axis. (**B**) Immunohistochemistry of PSP and PAPI & III in selected segments of the small intestine. PSP localizes to the duodenal part, predominantly to PANETH like cells. PAPI & III is more localized to enterocytes of the ileum.

To assess the potential for synthesis and release of PSP and PAP in the pancreas and intestine we first evaluated their distribution in rats. In healthy animals baseline levels in the pancreas were 90 ± 12 ng/mg (*n* = 19) for PSP and 6 ± 2 ng/mg (*n* = 20) for PAP I. The isoforms PAP II & III were hardly detectable (data not shown). In order to determine whether remote tissue injury causes an upregulation of these proteins, we measured the tissue contents of PSP and PAP I, II, & III in the intestinal tract. Ten segments of 10 cm intestine were harvested and proteins extracted. Figure 1A shows PSP at highest levels in the duodenum (segment 1, 2) with an intermittent rise in the jejunum. PAP I & III exhibited low levels in the proximal intestine (segment 1 to 6) and increased significantly in the ileum (segment 7 to 10). PAP II was not detectable in the entire intestine. The colon (segment 12) was negative for all proteins. While the stomach was positive for PSP and negative for all PAP isoforms, other organs such as kidney, liver, spleen, or lung were all negative for all tested proteins.

Immunohistochemical analysis of intestinal segments corroborated these findings (Figure 1B). While PSP is expressed in the duodenum, primarily in PANETH-like cells, PAP isoforms I & III localize mainly to enterocytes.

### Does PSP & PAP respond to remote lesions?

In order to answer the question whether organs producing PSP and PAP respond to remote injury we started a pilot experiment by inflicting a small lesion in the distal duodenum, followed by sampling immediately, 6, 12, and 24 hours later. We measured proteins from the lesion as well as from intact tissue 2 cm proximal and distal from the lesion. Supplementary Figure 1 demonstrates that both PSP and PAP strongly increased - at all sampled sites - with a peak at twelve hours, indicating that the synthesis of these proteins responds to damage in the vicinity.

In the next experiment we asked whether more distant lesions would induce an induction of PSP and PAP synthesis. For that purpose, we designed four groups of rat experiments receiving either a small lesion in the main duct of the pancreas, a lesion in the duodenum, a laparotomy only (sham operation), or a treatment mimicking anesthesia. Specimens were taken from the pancreas and the duodenum to determine synthesis and localization of PSP & PAP 6, 12, and 24 hours after surgery.

**Figure 2:**
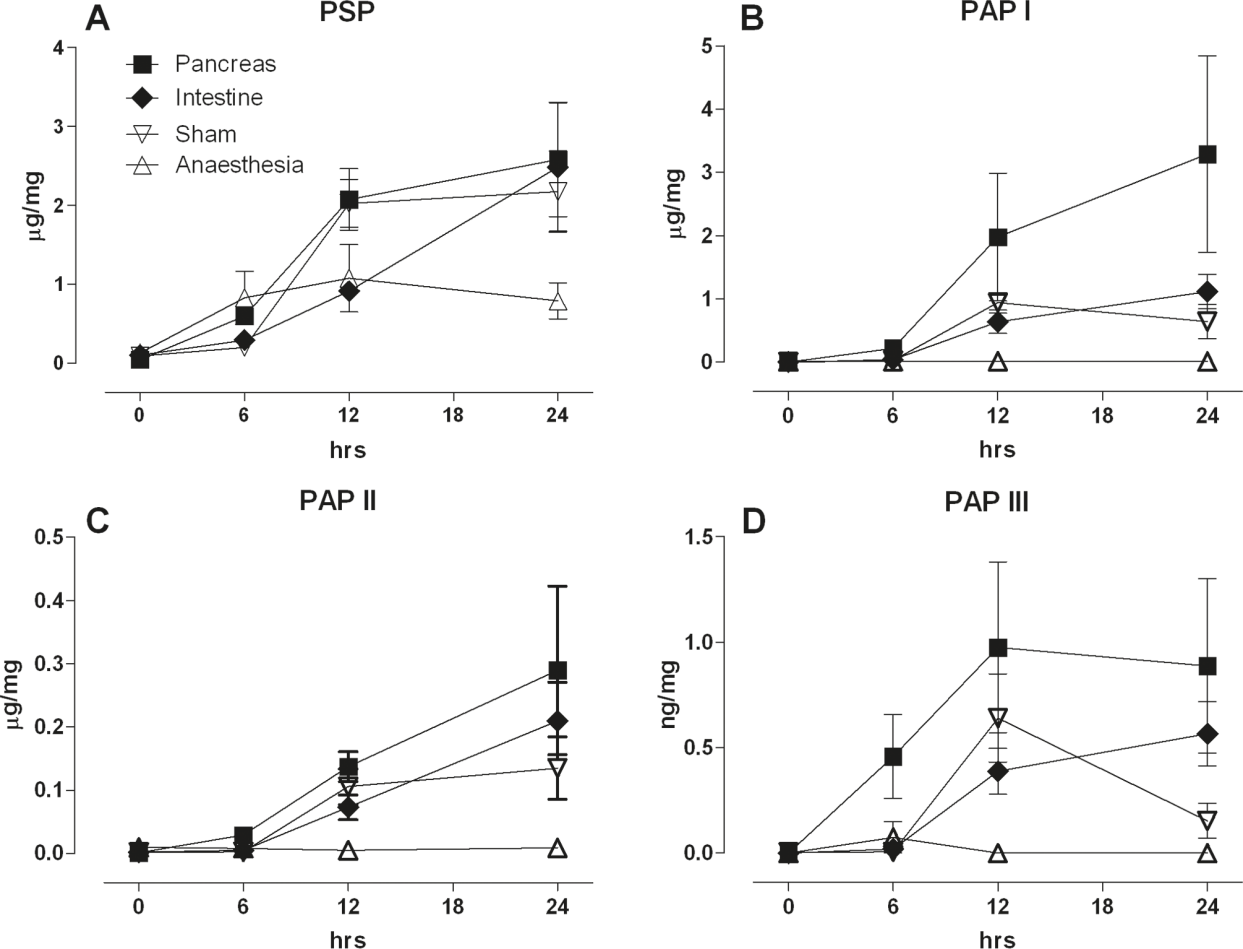
Response of the pancreas to a pancreatic ductal lesion (Pancreas), intestinal lesion (Intestine) laparotomy (Sham), or anaesthesia control (Anaesthesia) PSP and PAP isoforms were quantified immediately, 6, 12, and 24 hrs the operation. Tissue was homogenized, extracted, and the proteins determined for the different isoforms given in ng/mg total protein. Panel (**A**) PSP, (**B**) PAPI, (**C**) PAPII, and (**D**) PAPIII; (*n* = 5, mean ± SEM).

First, we looked at the pancreas in response to the various lesions. Not surprisingly, PSP increased over 24 hours in the pancreas in response to a pancreatic lesi on (Figure 2A). However, both a remote intestinal lesion as well as the sham laparotomy caused a similar increase in pancreatic synthesis of PSP. Furthermore, PSP in the pancreas was slightly elevated when the rat was subject to 20 minutes anesthesia only. PAP I, II & III reacted similarly to the various treatments with the exception of anesthesia which did not induce any increase in PAP isoforms (Figure 2B–2D).

**Figure 3:**
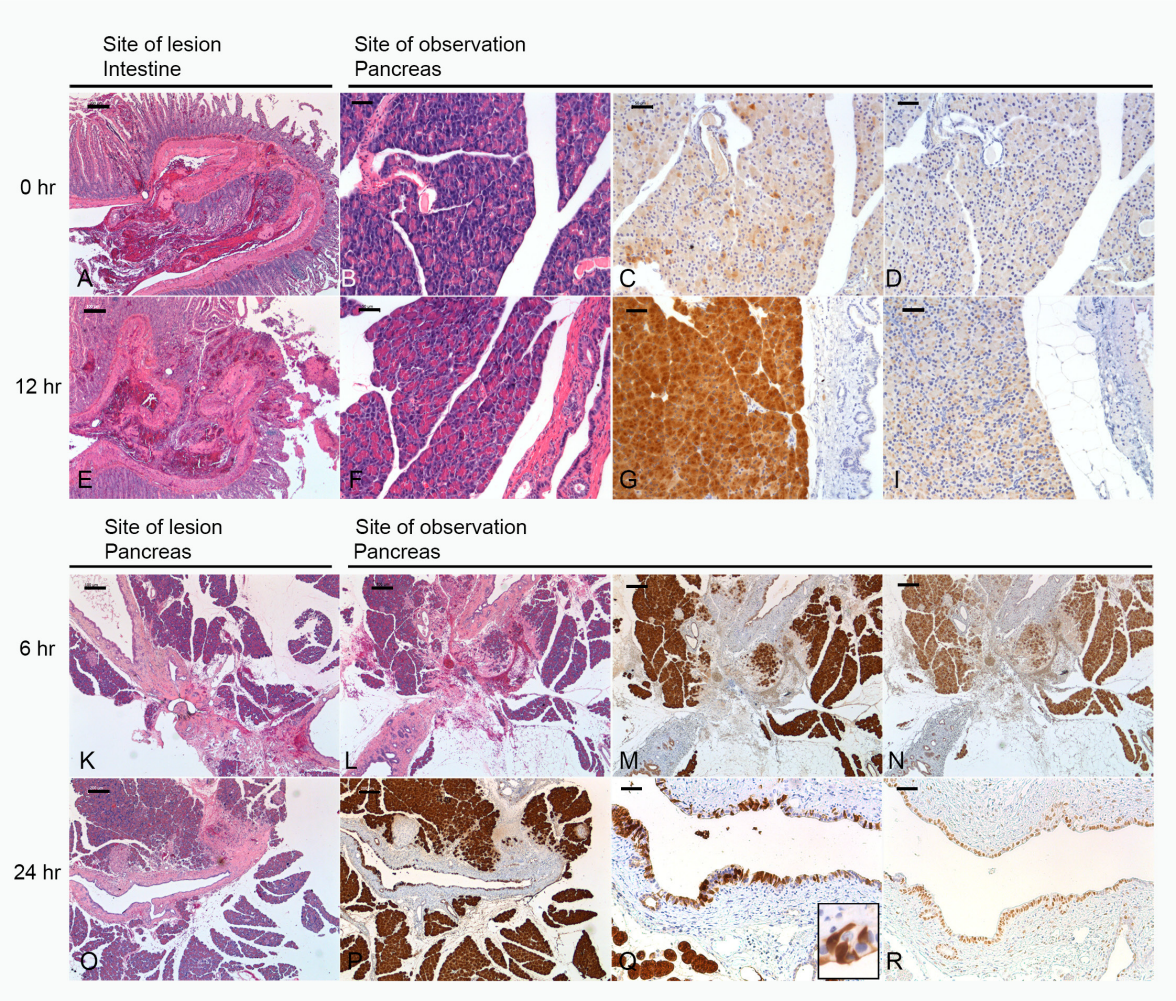
Morphology of lesions and their effect on the pancreas Upper panel: site of lesion is the intestine, morphology of lesion at 0 (**A**) and 12 hrs (**E**). (**B**, **F**) H&E section of pancreas at 0 and 12 hrs. (**C**, **D**) Immunohistochemical staining of PSP and PAPI at 0 hrs reflecting base-line levels. (**G**, **I**) Expression of PSP and PAPI 12 hrs after induction of a lesion. Lower panel: site of lesion is the pancreas. (**K**, **L**, **O**) H&E section demonstrating the morphology of the lesion at 0, 6, and 24 hrs. (**M**, **N**) immunohistochemistry of the pancreas localizing PSP and PAPI 6 hrs after the lesion. Note overlapping response. (**P**) PSP at 24 hrs in the vicinity of the lesion. Similar distribution pattern was observed for PAPI. (**Q**, **R**) Significant expression of PSP and PAPI in ductal cells, see also inset in Q. Bar = 200 mm (A, B, E, F, K, L, O, P), Bar = 50 mm (C, D, G, I, M, N, Q, R).

To corroborate our observations, we assessed pancreatic morphology and PSP and PAP I expression. When an intestinal lesion was incurred (Figure 3), the morphology of the pancreas did not change, however, PSP expression strongly increased from a punctate type to a strong homogenous staining (Figure 3G). PAP I, which is not detectable under baseline conditions, was only slightly visible after 12 hours (Figure 3D–3I). In case of a pancreatic lesion the reaction of PSP appeared much stronger in the vicinity of the lesion and also PAP I demonstrated a strong upregulation (Figure 3M, 3N, 3P). Furthermore, after 24 hr duct cells turned positive for both PSP and PAP I (Figure 3O, 3R).

### From these experiments we conclude that the pancreas responds to remote lesions

**Figure 4:**
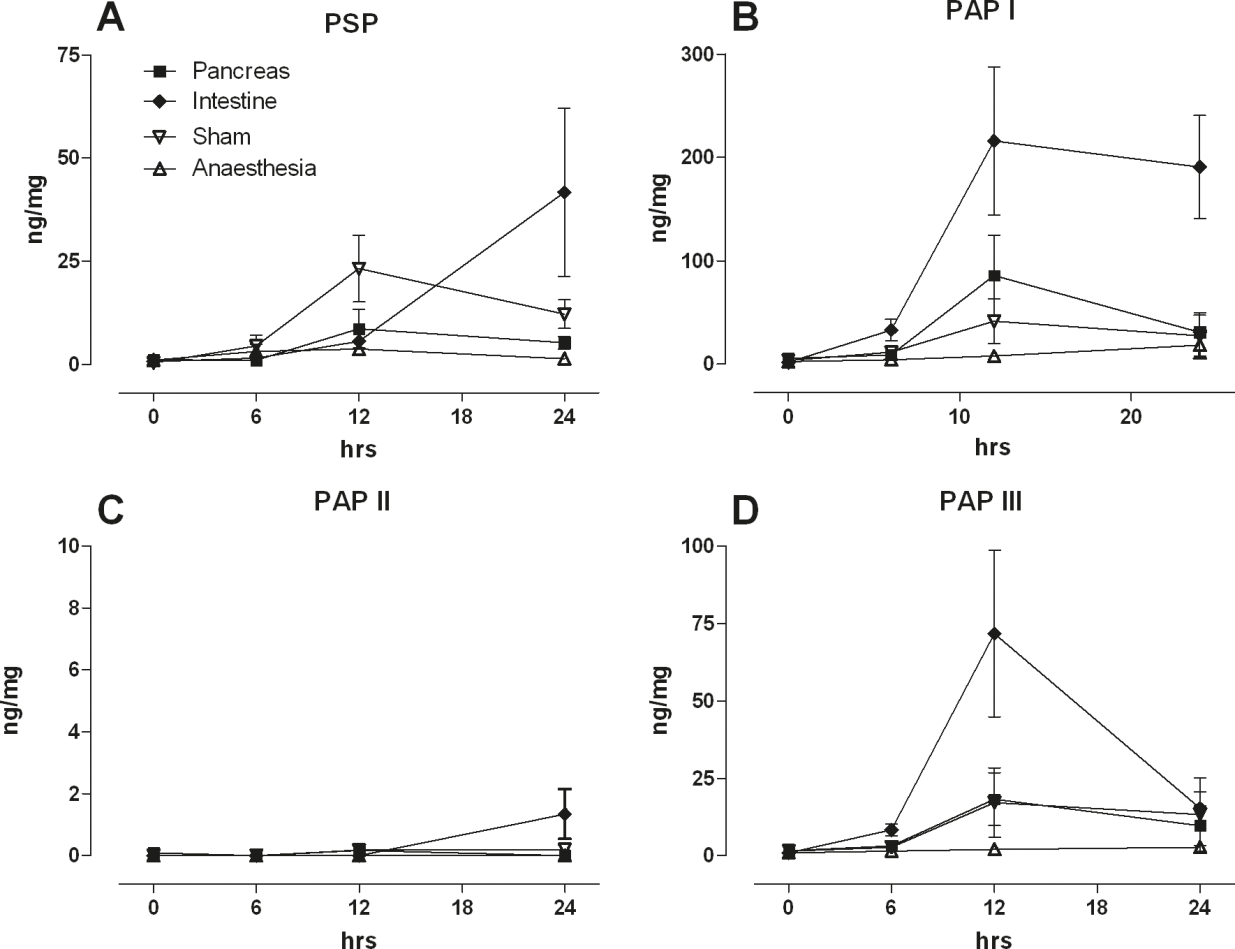
Intestinal levels of PSP, PAPI, II, III in response to a pancreatic ductal lesion (Pancreas), intestinal lesion (Intestine), laparotomy (Sham), or anaesthesia control (Anaesthesia) Tissue from a defined site was collected, homogenized, and quantified for (**A**) PSP, (**B**) PAPI, (**C**) PAPII, (**D**) PAP III. Note different axis levels for the various isoforms; (*n* = 5, mean ± SEM).

The same groups of animals were now assessed for changes in PSP & PAP expression in the intestine (Figure 4). PSP was increased in the sham, pancreatic, and intestinal lesion group while anesthesia did not invoke significant changes. The strongest increase was observed in the intestinal lesion group at 24 hrs indicating a local reaction. PAP I however, was strongly increased in the intestinal lesion group and to some extent in the pancreatic lesion group. Similarly, PAP III was strongly increased in the intestinal lesion group while pancreas and sham animals exhibited a moderate response. Taken together, both intestinal PSP and PAP seem to react preferentially to local damage and less to remote injury.

**Figure 5:**
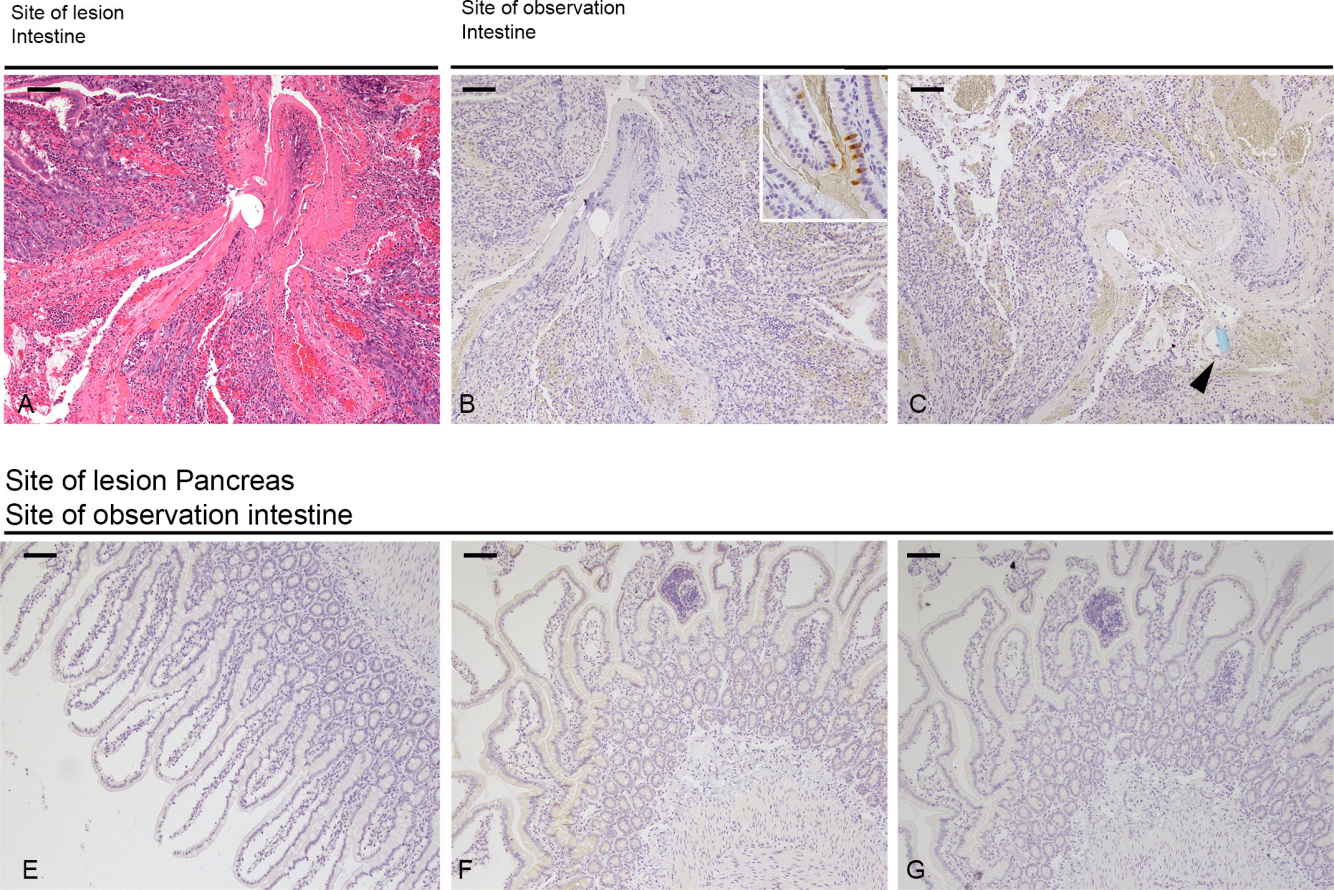
Morphology of intestinal lesion and tissue distribution of PSP & PAPI (**A**) H&E staining of the lesion at 0 hrs. (**B**, **C**) Immunohistochemistry at 0 hrs for PSP and PAPI. Inset in (B) demonstrates normal expression of PSP in PANETH like cells. Arrowhead points to the cross-section of a piece of blue suture material to tie the knot. Immunohistochemistry of intestinal sections after induction of a pancreatic lesion. Minimal expression of PAPI at 6 hrs (**E**) and 12 hrs (**F**) and PSP at 12 hrs (**G**).

Again we corroborated findings by immunohistochemistry. Since the overall contents of PSP and PAP in the duodenum is much lower, only moderate changes could be observed (Figure 5) for both proteins.

### Do distant septic lesions induce a pancreatic response?

**Figure 6:**
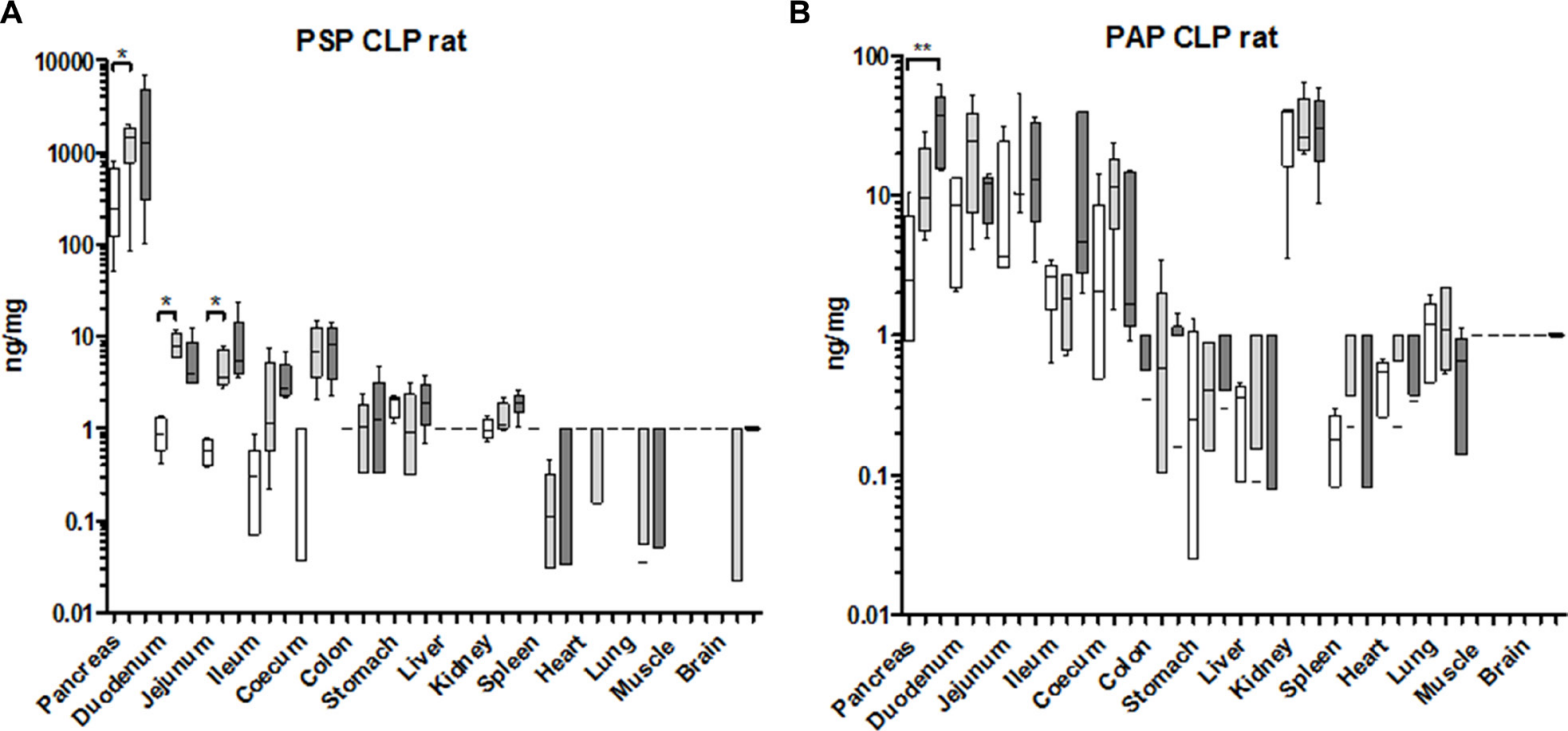
Organ-specific response of PSP and PAPI to a septic event in the rat. Organs from an experiment after caecum-ligation and puncture were analyzed for tissue contents (**A**) PSP, (**B**) PAPI. Organs tested are indicated at the bottom. Values are given in box-&-whisker-plots (median, min, max). Open boxes: base-line values, light grey boxes: sham-laparotomy, dark grey boxes: CLP. **p < 0.01 between untreated and CLP; *n* = 5.

Since it is already known from human trials that PSP reacts sensitively to septic conditions we included a model of sepsis: the caecum-ligation-puncture model (CLP) which releases gut-bacteria into the peritoneum. PSP contents of various organs were determined in untreated controls, in sham operated animals, and the CLP procedure five days after induction. In the pancreas, PSP was already increased due to a general surgical trauma (sham group) while the addition of ligation and perforation of the caecum (CLP) did not significantly enhance the response any further (Figure 6A). A similar reaction was observed in the duodenum, jejunum, ileum, and caecum while the colon did not respond. In contrast to PSP, PAP expression in the pancreas responded more pronounced when treated with the full CLP procedure, while the sham operation caused an intermediate increase. All other organs did not show a selective response in terms of PAP expression (Figure 6B). These findings are corroborated by transcript levels of PSP, PAP, and major cytokines (TNFa and IL-6) or the chemokine MCP-1 (Supplementary Figure 2).

We conclude that in rats PSP responds predominately to surgical trauma (laparotomy and mobilization) while PAP I is distinguishing between surgical trauma and infection/sepsis.

### How is the response of PSP and PAP in a mouse CLP model?

**Figure 7:**
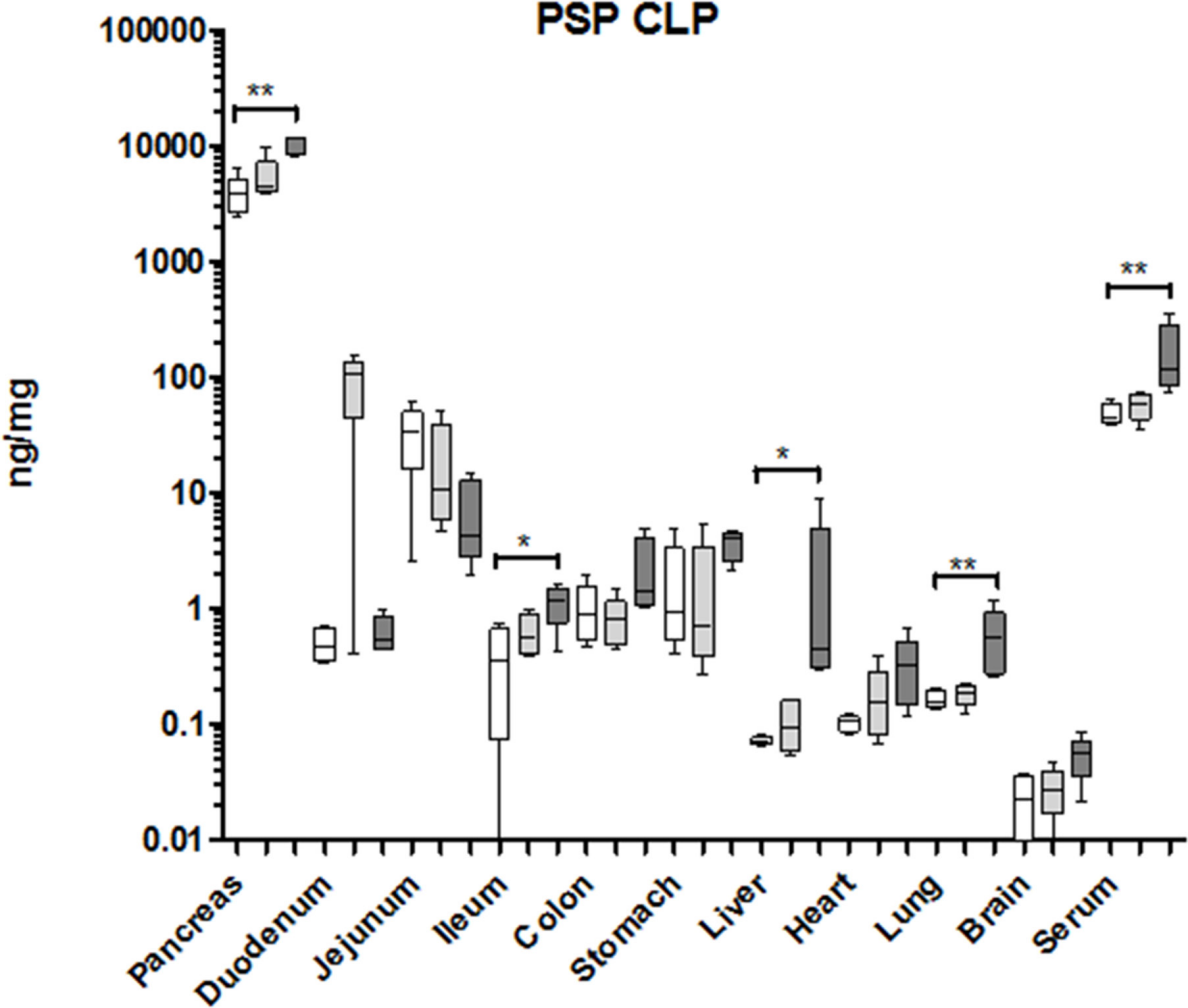
Organ-specific response of PSP to a septic event in the mouse Organs measured are indicated at the bottom. Values are given in ng/mg tissue or ng/ml serum, and shown as box-&-whisker-plots (median, min, max). Open boxes: base-line values, light grey boxes: sham-laparotomy, dark grey boxes: CLP. Significant differences between untreated and CLP treated mice: *p < 0.05, **p < 0.01; *n* = 5.

To evaluate whether similar control of PSP and PAP was active in mice we repeated the CLP experiments using a shorter period between induction of sepsis and sampling. In this model the pancreas reacted by a significant increase in PSP contents while the sham control was only slightly increased (Figure 7). In parallel serum PSP was significantly elevated in the sepsis group. In the intestine only the ileum demonstrated a significant PSP synthesis in the CLP group. Interestingly, the liver also reacted, albeit from very low levels to less than 1 ng/mg protein, a concentration of more than 105 times lower than the pancreas. To verify whether there was a systemic inflammatory response we measured IL-6 mRNA which was significantly upregulated in CLP mice while sham and controls did not display any changes (Supplementary Figure 3). We conclude that the pancreas senses the presence of a significant, infected lesion and is most likely the source of PSP release into the blood stream.

**Figure 8:**
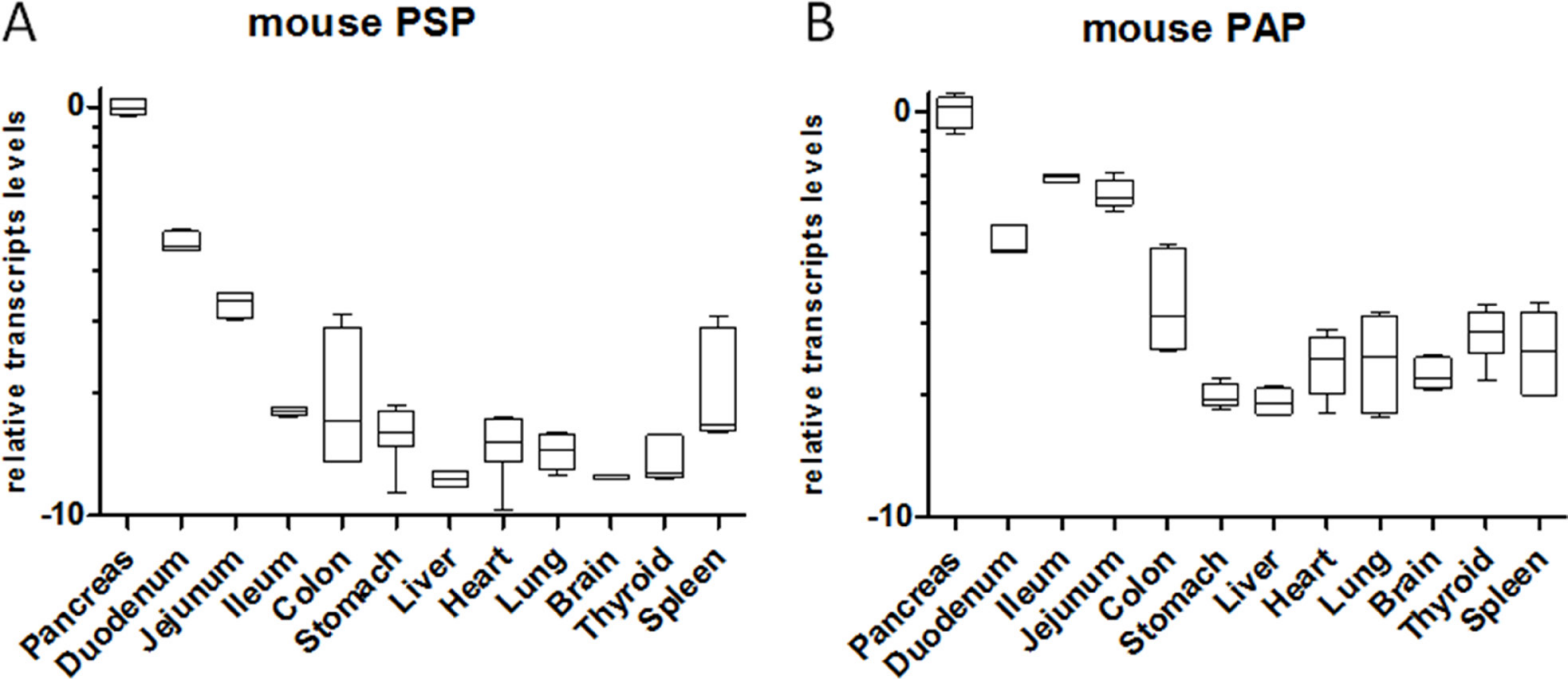
Relative transcript levels of PSP and PAP in the mouse at base-line in various organs RNA was extracted from healthy mice and transcripts were determined by qPCR. Organs are given at the bottom. All transcript levels were normalized relative to pancreatic transcript levels. Please note logarithmic x-axis; *n* = 5.

In addition, we also tested PSP and PAP mRNA levels in untreated mice. For PSP relative transcript levels were significantly higher in the pancreas (at least 105 fold) compared to any other tissue determined (Figure 8A). Similarly, mouse PAP (regIIIα) displayed the highest transcript levels in the pancreas compared to all other tested organs (Figure 8B).

### Which human organs produce PSP and PAP?

**Figure 9:**
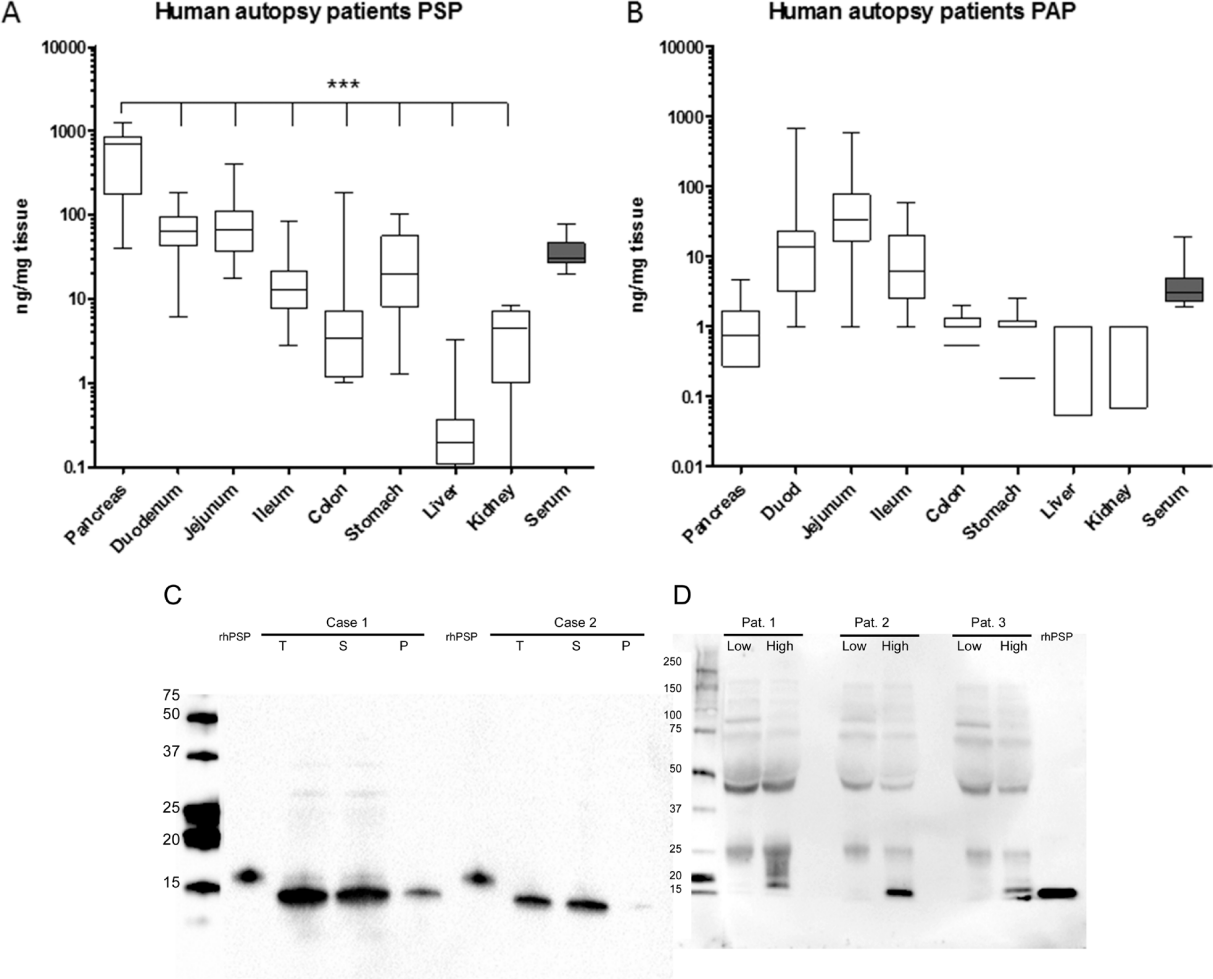
Tissue distribution of PSP (**A**) and PAP (**B**) in human autopsy cases after unnatural death. Organs indicated below the x-axis were analyzed by ELISA. PSP and PAP contents are expressed as ng/mg tissue protein. Pancreatic PSP content was significantly higher (*p* < 0.001) than any other tissue. Serum values are indicated in ng/ml. *N* = 16 cases. (**C**) Western blot analysis of pancreatic extracts for the presence of PSP in two autopsy cases. Extraction was performed as indicated in material and methods. T = total protein loaded. S = soluble protein fraction, P = insoluble (pellet) fraction. Recombinant human PSP was loaded as indicated. (**D**) Western blot analysis of serum PSP of three patients before surgery and after surgery when PSP contents were highest. Size of marker on the left, 2 ng recombinant PSP on the right.

We have demonstrated so far that PSP increases in non-pancreatic, general inflammation and sepsis, hence we concluded that PSP responds to systemic triggers. Since PSP and PAP are also synthesized by other intestinal organs in mice and rats, we wanted to evaluate whether in humans they are primarily produced by the pancreas or whether other organs could contribute significantly to the release of PSP and PAP. Therefore, we quantitatively analyzed the contents of PSP and PAP in human organs from 16 autopsy cases. Figure 9 shows the contents of both proteins in the intestine, stomach, liver, and kidney. Importantly, the human pancreas was shown to produce PSP at more than an order of magnitude higher than any other organ (Figure 9A). Immunohistochemistry of PSP and PAP in intestinal organs support these determinations despite visible degradation, particularly in the pancreas (Supplementary Figure 4). Serum levels were quite similar to our normal values found in healthy subjects. In contrast, PAP was predominantly produced in the small intestine while the levels in the pancreas were quite low (Figure 9B).

Furthermore, PSP was detected in homogenates from pancreata of two selected cases by western blot analysis. Compared to recombinant PSP at 16 KDa, most of the extracted PSP had an approximate molecular weight of 14 KDa which is a known cleavage product lacking the 11 terminal amino acids. In contrast to the known propensity of rodent PSP to form insoluble fibers after cleavage, the extracted PSP was soluble (Figure 9C). So far the molecular forms of PSP have mainly been characterized in pancreatic juice. We therefore analyzed serum from three patients that developed septic complications postoperatively (Figure 9D). On western blot analysis preoperative serum did not show any bands while 16kDa bands were clearly visible in serum during sepsis.

We also explored the potential of mRNA-levels despite the susceptibility to degradation in dead tissue. Surprisingly, when PSP-mRNA was detected by quantitative PCR we found levels similar to ‘fresh’ pancreatic biopsy tissue (Supplementary Figure 5) plotted as ΔCt relative to 18S RNA. PSP-mRNA in the small intestine was much lower, reflecting its corresponding protein levels. PAP in contrast, was most abundant in the small intestine, primarily in the jejunum. The unexpectedly high levels of PSP in the pancreas led us to look at the 18S RNA to which transcript levels are normalized. Indeed, Ct values indicated that in autopsy cases the retrieval of RNA yields only about 1% of fresh biopsy indicating advanced degradation.

From the bio-distribution in ‘healthy’ subjects (not septic) we conclude that the major source of PSP is from the pancreas while PAP is mainly produced by the intestine.

## DISCUSSION

Here we show the response of the pancreas to local and systemic injury with respect to production of PSP and PAP, two proteins known to be highly induced during pancreatitis. We observed increased production and secretion when a lesion was induced remote from the pancreas. This supports the concept that this organ, similar to the liver can act as an acute phase organ. Furthermore, we also show the quantitative distribution of these proteins in healthy human organs.

We and others have shown in several clinical studies that PSP/reg is significantly upregulated in patients with septic complications. Since it has been known for a long time that PSP is not only produced in the pancreas but also in intestine and stomach it was never clear where it was released from during a septic episode. We therefore set out to clarify the relative distribution of PSP and PAP in rodents and human subjects and tried to evaluate the origin of PSP in experimental models. It has to be stated up front that an absolute proof for which organ is primarily secreting these proteins (during sepsis) is currently not possible as the complexity of promoters driving tissue specific expression (e.g. in pancreatic acinar cells versus intestinal PANETH cells) has not been clarified yet, and hence genetic (knock-out/knock-in) models may not give an unambiguous answer.

Recent evidence from clinical studies indicates that PSP is highly increased and secreted into blood at the onset of sepsis [16]. Normally PSP is secreted into the pancreatic duct system along with zymogens to promote digestion. In case of a pancreatic injury PSP is also increased and, presumably due to tissue damage, is released into the interstitium and blood. This appears to be a passive effect based on damaged cells which is presumed to be the reason for elevated serum amylase and lipase levels during pancreatitis. Upregulation of these enzymes by enhanced synthesis is not observed, rather the opposite: there is a tendency for a reduced amylase/lipase content during pancreatitis. In contrast, PSP synthesis is strongly increased in acinar cells and hence explains the appearance of significant serum levels.

These observations lead us to believe that beyond passive release, PSP is actively stimulated by factors that may be local or systemic. As such, it is known that PSP can be induced by cytokines, e.g. IL-6. The function of PSP has been controversial, particularly since it was assumed for a long time that PSP is a secretory protein released into the duct and hence would exert its function in the intestine. These studies revealed a potential to inhibit stone formation, as a regenerative factor for β-cells, or as a protein promoting aggregation of intestinal bacteria. A discussion of these functions is beyond the focus of this report; however, the function of PSP in blood still remains open. We have demonstrated that neutrophilic granulocytes can be activated by PSP [16]. Yet it remains uncertain whether there is a direct interaction with a membrane-bound receptor as binding of PSP required a high concentration. This indicates that PSP might require a binding partner that subsequently induces activation.

While responsive to septic incidents, other triggers may also increase the presence of PSP in blood. As an example, it has been reported that diabetes [27, 28], renal failure and liver cirrhosis [29] may also induce elevated levels of PSP.

Quantitative data on the tissue distribution of PSP and PAP has not been available until now and, although merely descriptive, these data might give reference for future studies to evaluate potential sources of these proteins under physiological and pathological conditions. As expected PSP levels were highest in the pancreas in humans and rodents and increased after experimental septic events in mice and rats. In the intestine a consistent pattern was observed in the three species tested. This points to a conserved function in the gastrointestinal tract. Also, there was a selective increase during sepsis in segments of the small intestine while other organs did not react. This reaction is not only local but induces a systemic response that includes upregulation of PSP in ‘remote’ organs. Our initial experiments tested whether small lesions affecting one part of an organ only would induce systemic response and synthesis of PSP and PAP in another organ. Particularly, we assessed whether the pancreas could be a sensor for such lesions and thereby act as an organ centrally releasing PSP as part of an acute phase response. Indeed, the pancreas contains the highest amount of PSP, is activated to release PSP, and senses organ damage, particularly when associated with septic complications.

Our quantitative findings corroborate numerous reports on localization of PSP in individual organs where PSP has been described [11–14]. Also PAP (regIII) determinations are quite consistent with previous reports [14]. One caveat is the existence of multiple isoforms demonstrating selective regulation. This is best exemplified in the small intestine of the rat where PAP II is not expressed while PAP I & III are clearly and consistently expressed in certain segments. Incidentally, PAP II is the only known isoform that does not form insoluble fibrils after tryptic cleavage of the N-terminal peptide [30]. We assume that localization and function are tightly correlated; hence, PAP II might exert a different role than the other isoforms in a non-redundant way. A further example is mouse regIIIγ, which appears to be involved in bacterial colonization [31].

In summary, we provide quantitative tissue specific data of PSP and PAP isoforms in humans, rats, and mice. Baseline production of PSP is several magnitudes higher in the pancreas than any other organ while PAP is predominantly expressed in the small intestine. Under systemic stress both PSP and PAP are predominantly increased in the pancreas of rats and mice.

## MATERIALS AND METHODS

### Reagents

All reagents were of analytical grade. If not otherwise stated they were purchased from SIGMA-Aldrich, Buchs, Switzerland.

### ELISA

Enzyme linked immunosorbent assays (ELISAs) detecting PSP & PAP isoforms in humans, rats, and mice were performed with antibodies produced by our lab as previously described [15, 16, 32, 33]. In brief, the open reading frame was cloned into a *Pichia pastoris* vector in frame with a signal directing the polypeptide to be secreted into medium [34]. The positive clones were amplified and tested for efficiency of secretion. One clone was further expanded for production of the polypeptide. Concentration and purification was performed on an Akta System (Pharmacia, Dübendorf Switzerland) using a cation exchanger with a lithium chloride/pH gradient.

For generation of antibodies, the purified polypeptides were injected into guinea pigs and rabbits using Friend’s complete and incomplete adjuvans. Animals reaching a high titer (usually 1:100′000 dilution to detect 0.5mg/ml coated on an ELISA plate) were then terminally bleed. Subsequent purification of IgG was performed on proteinA columns (Pharmacia, Dübendorf, Switzerland). This procedure was followed for the antibodies used in the study.

The sandwich ELISA followed a common procedure: guinea pig IgG was coated over night, washed and blocked with TBS/BSA, followed by incubation with sample or standards. Subsequently the secondaryrabbit antibody was incubated, followed by a phosphatase-conjugated anti-rabbit antibody (SIGMA, Buchs, Switzerland). The plate was developed with a phosphatase substrate and OD 405 measured on a Tecan ELISA Reader.

### Immunohistochemistry

Paraffin embedded formalin-fixed tissue was sectioned at 3mm. To deparaffinize, the sections were carried through an increasing alcohol series. For antigen demasking the sections were boiled in citrate for 20 minutes and subsequently allowed to cool. The sections were then blocked overnight at 4°C prior to incubation with the primary antibody in a humidified chamber. Detection of the primary antibody followed according to the manual of the ABC Vector stain detection kit (Vector Laboratories, Burlingame, Ca, USA). The sections were counterstained with Haemalaun and covered by a glass slip. The formalin-fixed human tissue was deparaffinized in a PT-link and stained on an Autolink from Dako (Denmark). Antibodies were used as described [15, 16, 32, 33].

### Isolation of RNA and RT-PCR

RNA from pancreata and other organs followed the procedure described earlier [15]. Total RNA was quantified by Nanodrop 2000 (Thermo Scientific, Zug, Switzerland) and a quality control was performed by a Bioanalyzer 2100 (Agilent, Basel Switzerland). Subsequently, 1 μg total RNA was reverse transcribed and used for real-time polymerase chain reaction (PCR) as described previously [15, 30] or with a newly established method using the Precellys®24 Dual homogenizer (VWR, Dietikon, Switzerland) with MagNA Lyser Green Beads from Roche Applied Science (Basel, Switzerland). In brief, a small piece of snap frozen tissue was transferred to a tube containing beads, 650 ml of Lysis buffer (Qiagen, Hilden, Germany) was added and the tube immediately homogenized in a Precellys shaker for 30 seconds at 6000 rpm. RNA was extracted following the Qiagen RNeasy Mini Kit extraction protocol with an on column DNase digestion. Purified RNA was reverse transcribed into cDNA using qScript™ cDNA SuperMix of Quantabio (Beverly, MA, USA) according to the manufacturer’s protocol. Quantitative PCR was then performed on an ABI 7500 FAST sequence detection system (Applied Biosystems, Zug, Switzerland).

### Animal studies

All animal studies were executed according to Swiss animal protection laws. Protocols were subjected to review by the Veterinary Offices and approved by the Ethics committee of the Canton of Zurich. The study on mice was performed on biopsies purchased from PHENOS (Hannover, Germany).

### Rat I (injuries to pancreas and intestine)

Male rats (Sprague Dawley) were purchased from Harlan (Horst, the Netherlands) and allowed to adapt for two weeks in our SPF-facility with access to food and water *ad libitum*. Prior to the operation, rats were starved overnight. Following induction of anesthesia by Sevoflurane inhalation, the animals were randomly sorted into one of four groups: 1) anesthesia only (anesthesia group), 2) median laparotomy only (sham group), 3) induction of a small lesion of the major pancreatic duct (pancreas group), or 4) induction of a small lesion of the small intestine (intestine group). The length of procedure in group 3 and 4 was approximately 20 minutes, therefore the animals of group 1 and 2 were also kept under anesthesia for a similar time period. All procedures were performed under a sterile hood on a heating plate (37°C). To harvest plasma and tissue rats were sacrificed and small pancreatic specimens were excised from the site of injury and from a site remote to the damage (6, 12, and 24 hrs after surgery). Intestinal tissue was directly collected from the lesion site as well as 2 cm proximal and distal of the lesion. All tissue specimens were frozen at -80°C. Animal tissues were used to quantify PSP and PAP by isoform-specific ELISA and to detect the proteins by histology.

### Rat II (CLP-Model)

Induction of sepsis in rats was performed according to standard procedures for the caecum ligation and puncture (CLP) model as reported in [31]. Briefly: Rats were anaesthetized with isoflurane. Following laparotomy the caecum was localized, mobilized, and briefly moved (sham group) or ligated by a 5.0 suture (CLP group). The caecum was then punctured with a 19-G needle (CLP group). The laparotomy was closed by a suture and the rats were then allowed to wake up on a heating pad. All rats were sacrificed five days after surgery and animal plasma and organ tissues (all abdominal organs, heart, lungs, muscle, brain) were used for analysis of PSP and PAP (ELISA, PCR and histology).

### Mouse CLP Model

The CLP model in mice (C57BL/6) was performed in accordance with the rat CLP experiments. Tissue was harvested after 6 hours.

### Human study (protein expression in healthy tissues and in plasma)

Organs of sixteen cases succumbed to unnatural death were collected by the medical examiner. Tissue samples were immediately frozen in liquid nitrogen and stored at −80°C. Dissection was performed 8 ± 2 hours after death. The cases appeared to be healthy prior to death. Analysis of material from human subjects by the medical examiner (C.P., medico-legal autopsies) was performed as requested by the inquiring authorities, respecting guidelines of the Canton de Vaud, Switzerland. The samples collected for this study are routinely collected during autopsy for histological purposes. No ethical approval was necessary to collect samples and perform analyses in the collected cases.

In the last part of this study serum from three patients that developed septic complications was used for PSP quantification, approved by the Kantonale Ethikkommission Zurich and registered under https://clinicaltrials.gov/ (NCT01258179).

### Statistical methods

For most experiments the group size was five animals. Comparison between two groups was performed by Student’s *t*-tests, whereby *p* < 0.05 was considered statistically significant. The distribution within a group was assessed and checked for Gaussian distribution. For comparisons between three groups one-way ANOVA was performed with Dunnetts post-hoc test to identify differences between individual groups. The statistical software GraphPad Prism (San Diego Ca, USA) was used for all calculations.

## SUPPLEMENTARY MATERIALS FIGURES


