# Supplementary material for: The pancreas responds to remote damage and systemic stress by secretion of the pancreatic secretory proteins PSP/regI and PAP/regIII
Source: Oncotarget. 2017 Mar 16;8(18):30162–74. doi: 10.18632/oncotarget.16282 (PMC5444734; doi:10.18632/oncotarget.16282)
Supplement: Supplementary file 1 [file oncotarget-08-30162-s001.pdf]

The pancreas responds to remote damage and systemic stress by secretion of the pancreatic secretory proteins PSP/regI and PAP/regIII

Supplementary Materials

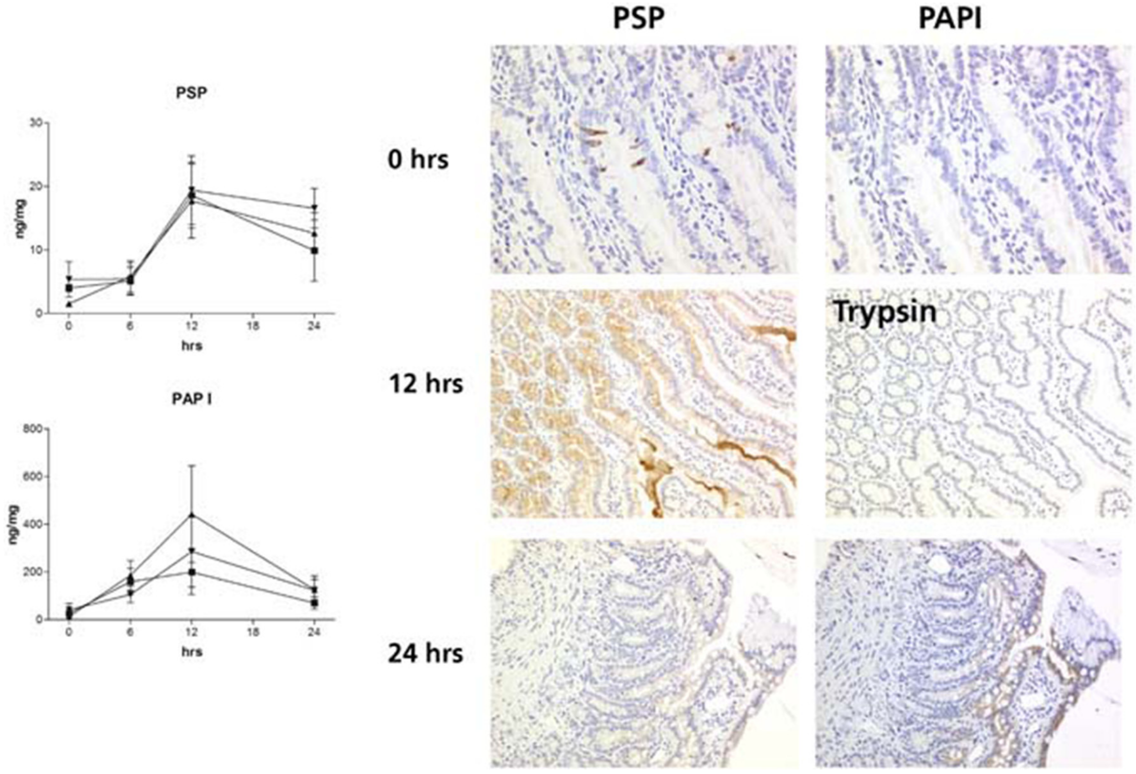

Supplementary Figure 1: PSP and PAP levels 0–24 hrs after induction of an intestinal lesion in the rat. A small incision into the intestinal mucosa was introduced and immediately closed by a suture. Samples were taken at the site of lesion (■), 2 cm proximal (▲), and 2cm distal to the lesion (▼), homogenized and quantified by ELISA for the presence of PSP (**A**) and PAPI (**B**). *N* = 5, mean ± SEM. Immunohistochemical detection of PSP and PAPI at 0, 12 hrs and 24 hrs after induction of a lesion. Trypsin: same section as in left panel but stained with an antibody recognizing trypsin.

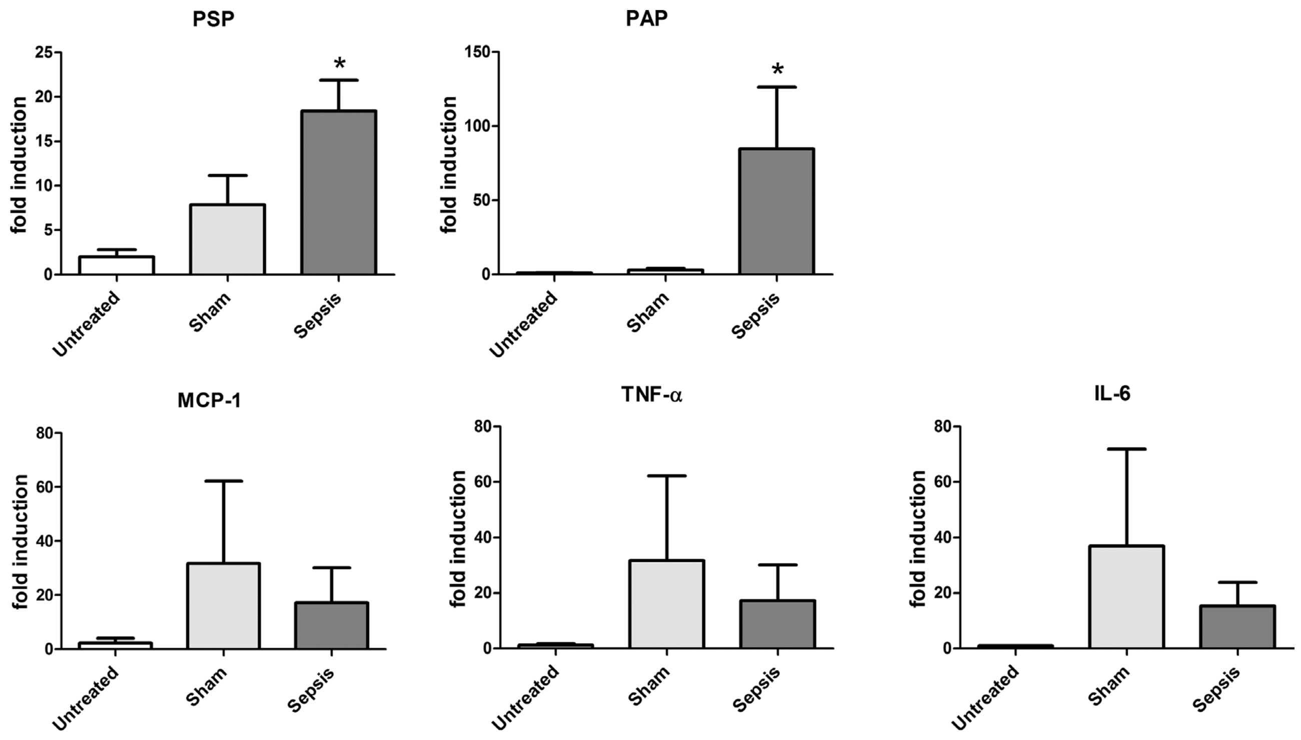

Supplementary Figure 2: Transcript levels of various genes in untreated, sham operated, and CLP rat pancreas. Note that most transcript levels are already increased after sham operation. Genes indicated above the graphs. $P < 0.05$ versus untreated. $N = 5$ animals per group.

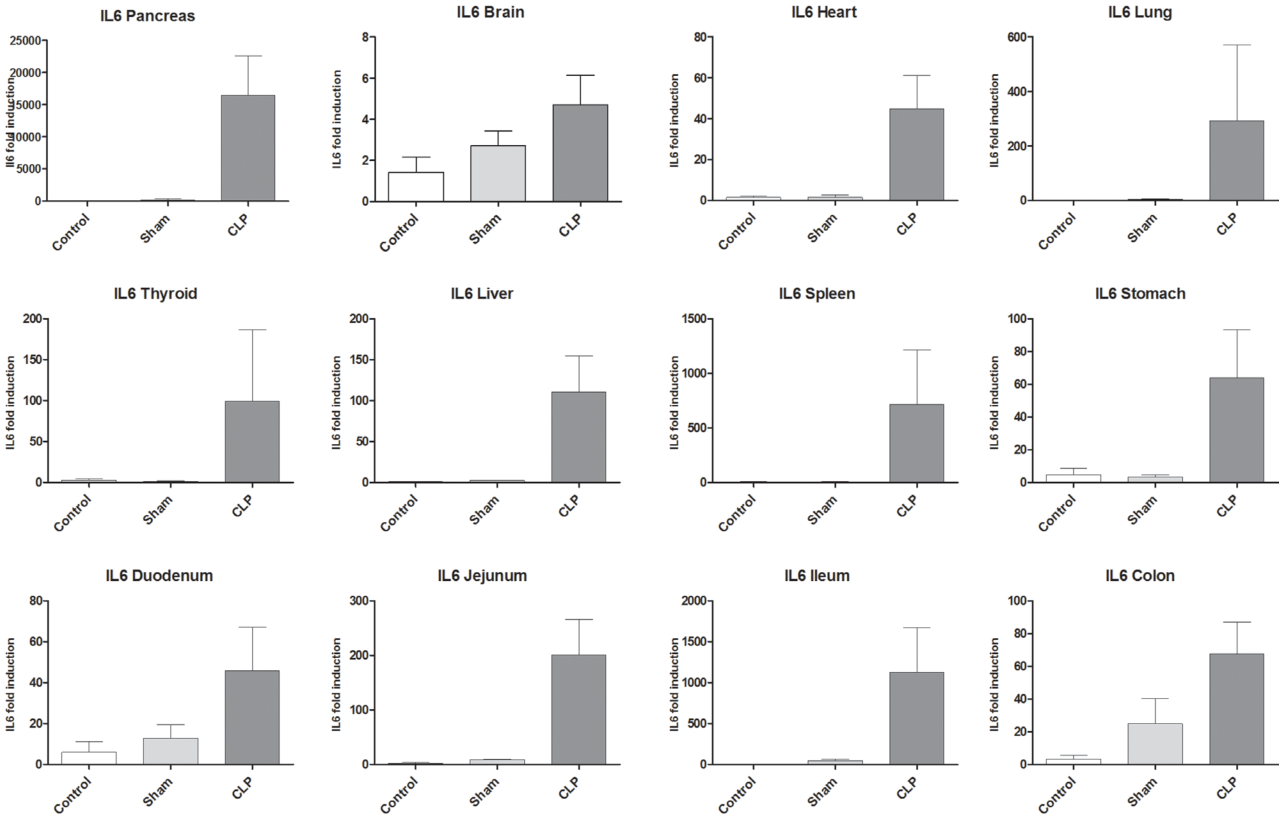

Supplementary Figure 3: IL-6 transcript-levels in various organs after untreated, sham, and CLP in mice. For each organ the level of the untreated group was normalized to one. Organs indicated above the respective graph. *N* = 5 animals per group.

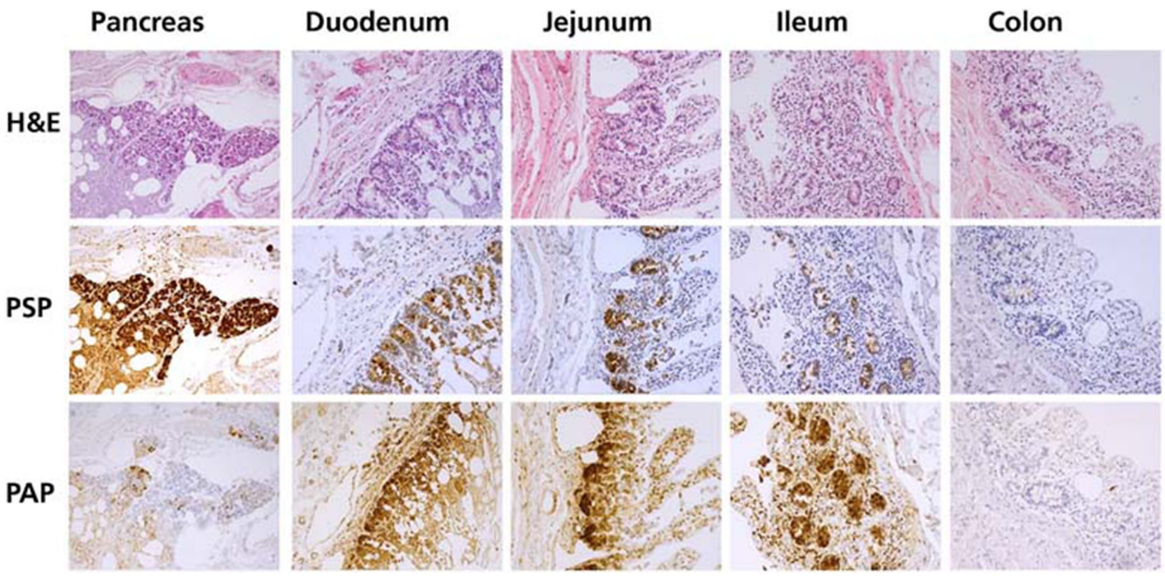

Supplementary Figure 4: Histological and immunohistochemical assessment of various intestinal organs. Top row: H&E staining. Middle row PSP, lower row PAP. Organs are indicated at the top.

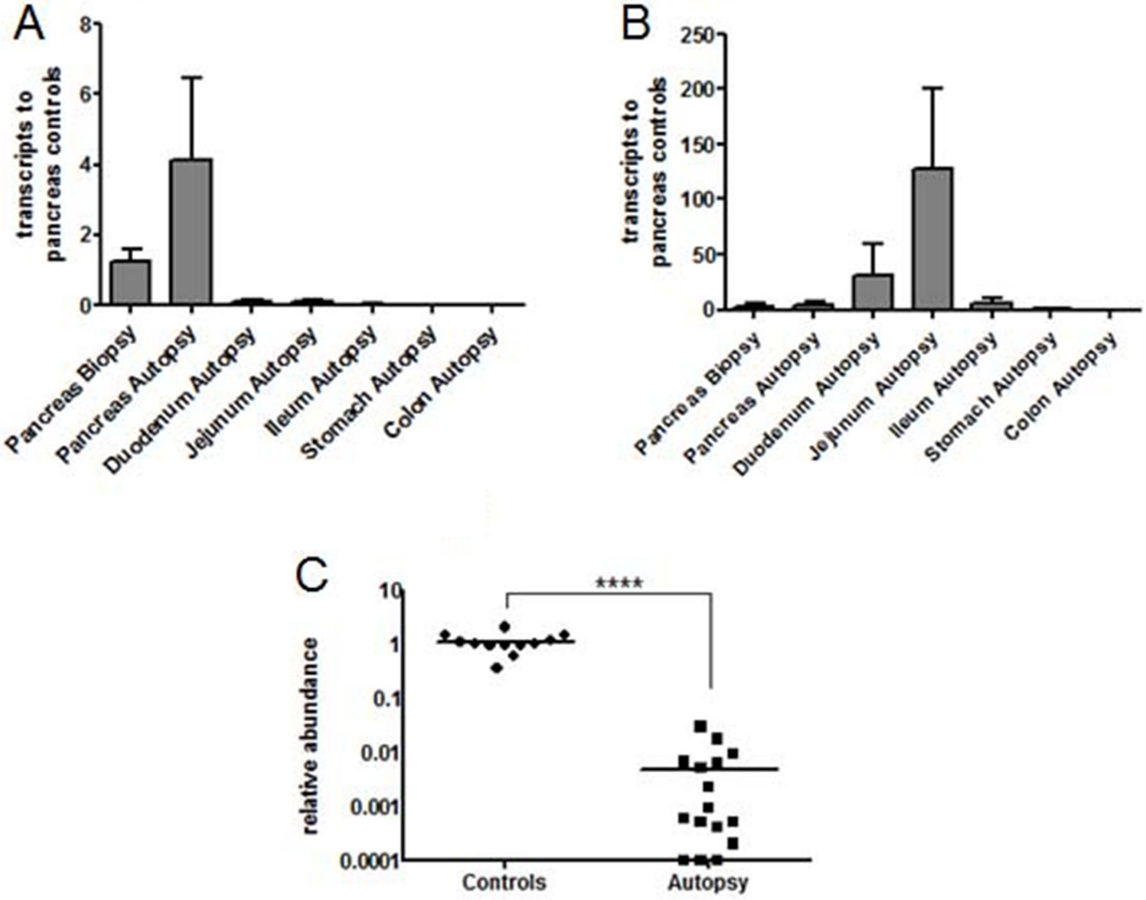

Supplementary Figure 5: Detection of transcripts in human pancreas and various organs. Tissue was extracted and analyzed by qPCR for PSP (**A**) and PAP/regα (**B**) in biopsies from surgically resected specimen (pancreas biopsy) and from autopsy specimen. Relative abundance normalized to 18S RNA was compared to biopsy RNA from resected specimen. (**C**) Relative abundance of 18S RNA in surgically (♦) resected compared to autopsy specimens (■). Note the difference of two orders of magnitude $p < 0.0001$.
